# Giant and irregular pituitary neuroendocrine tumors surgery: comparison of simultaneous combined endoscopic endonasal and transcranial and purely endoscopic endonasal surgery at a single center

**DOI:** 10.1186/s41016-025-00389-4

**Published:** 2025-02-03

**Authors:** Jun Fu, Wenwei Luo, Chunlin Zhang, Zhicheng Wang, Wenjian Fan, Yuanxiang Lin, Dezhi Kang, Jianping Song, Changzhen Jiang, Xiaorong Yan

**Affiliations:** 1 Department of Neurosurgery, Neurosurgery Research Institute, The First Affiliated Hospital, Fujian Medical University, Fuzhou, Fujian 350005 China; 2 Department of Neurosurgery, National Regional Medical Center, Binhai Campus of the First Affiliated Hospital, Fujian Medical University, Fuzhou, Fujian 350212 China; 3 Department of Neurosurgery, Nanping First Hospital Affiliated to Fujian Medical University, Nanping, Fujian 353000 China; 4 Department of Neurosurgery, Huashan Hospital, Shanghai Medical College, Fudan University, Shanghai, 200040 China; 5 National Center for Neurological Disorders, Shanghai, 200040 China; 6 Neurosurgical Institute of Fudan University, Shanghai, 200040 China; 7 Shanghai Clinical Medical Center of Neurosurgery, Shanghai, 200040 China; 8 Shanghai Key Laboratory of Brain Function Restoration and Neural Regeneration, Shanghai, 200040 China; 9 Research Unit of New Technologies of Micro-Endoscopy Combination in Skull Base Surgery (2018RU008), Chinese Academy of Medical Sciences (CAMS), Shanghai, 200040 China

**Keywords:** Pituitary neuroendocrine tumors, Combined approach, Endoscopic endonasal surgery, Transcranial surgery

## Abstract

**Background:**

Surgical management of giant and irregular pituitary neuroendocrine tumors (GIPitNETs) presents a significant challenge in neurosurgery. While endoscopic endonasal surgery (EES) is a widely used approach for PitNETs, GIPitNETs with extensive intracranial extension pose challenges for purely EES. We use simultaneous combined endoscopic endonasal and transcranial surgery (CECS) for the treatment of this type of tumor. Currently, there is limited research comparing CECS to EES for GIPitNETs. This study aims to compare the efficacy and short outcome of CECS and purely EES in the management of GIPitNETs to better understand the advantages and limitations of each surgical approach.

**Methods:**

The data of GIPitNETs patients who underwent surgery between March 2018 and May 2023 at a single center were retrospectively reviewed. All included cases were divided into CECS and EES groups according to the treatment modality received. The baseline characteristics and tumor imaging features of patients were compared between the groups, as well as surgical results, perioperative complications, and last follow-up outcomes.

**Results:**

A total of 50 patients met the inclusion criteria, with 27 undergoing CECS and 23 EES. CECS achieved a significantly higher GTR rate compared to EES (66.7% vs. 13.0%, *p* < 0.0001). CECS had longer operation times and hospital stays, but both approaches had similar rates of complications, including intracranial infection, CSF leakage, new pituitary dysfunction, postoperative diabetes insipidus, and vascular infarction. CECS reduces the risk of postoperative bleeding. Tumor recurrence and reoperation were significantly more common in the EES group.

**Conclusions:**

CECS is a safe and effective surgical approach for GIPitNETs, leading to higher rates of GTR, comparable complication rates, and reduced risk of postoperative bleeding when compared to purely EES. EES was associated with more tumor recurrence. Further long-term follow-up data is needed to validate these findings.

**Supplementary Information:**

The online version contains supplementary material available at 10.1186/s41016-025-00389-4.

## Background

**Fig. 1:**
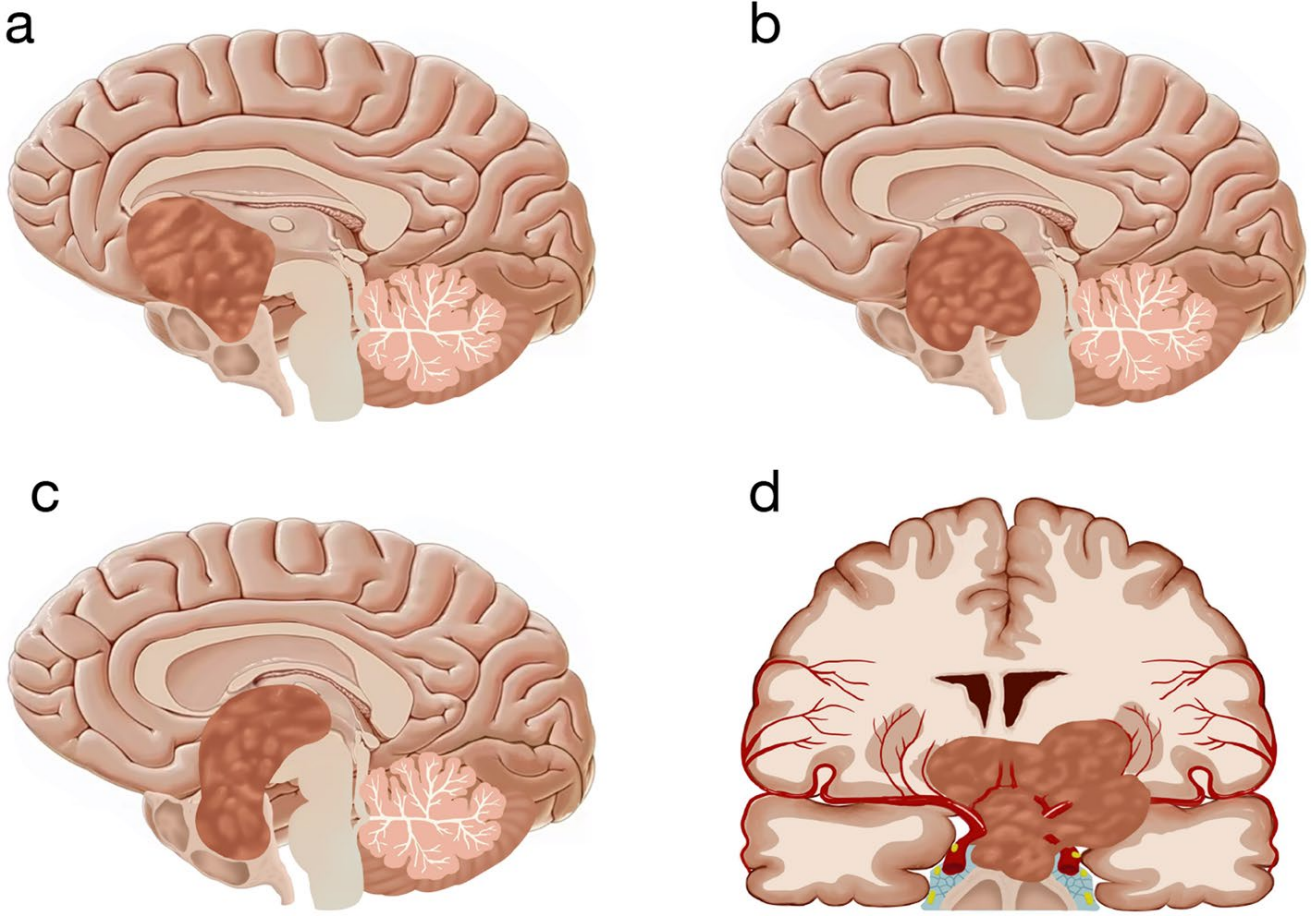
Key GIPitNET configurations include the following: **a** anterior extension, **b** posterior extension, **c** superior extension, **d** lateral extension, and vascular encasement

The surgical treatment strategy for pituitary neuroendocrine tumors (PitNETs) has always been a focal point in the field of neurosurgery. In recent years, endoscopic endonasal surgery (EES) has been widely used for treating giant pituitary adenomas for treating PitNETs [1–4]. However, for giant and irregular PitNETs (GIPitNETs), especially those tumors growing beyond the sella, extending upwards into the intracranial space, potentially breaching the diaphragm, and/or affecting nearby neurovascular structures, as depicted in Fig. 1. The tumor presents a significant intracranial extension that is outside the visibility and maneuverability of the endoscopic endonasal route so that purely EES may face challenges [5, 6]. Although angled endoscopes (30–70°) can provide limited visualization, the use of surgical instruments becomes difficult [7–9]. Just like reported in some articles, it is almost impossible to remove a GIPitNETs totally [10, 11]. Therefore, innovative strategies are required. In recent years, the simultaneous combined endoscopic endonasal and transcranial surgery (CECS) has been reported in the treatment of these cases [8, 12]. Currently, there is currently limited research on the CECS. Comparative studies and meta-analyses on the efficacy and complications of the CECS and purely EES for GIPitNETs are lacking sufficient data.

The aim of this preliminary report is to compare the efficacy and safety of the CECS with the purely EES in the management of GIPitNETs to define the benefits and limitations of the various surgical approaches.

## Methods

We designed a retrospective observational single-center cohort study of all CECS or EES procedures performed for GIPitNETs between March 2018 and May 2023 at The First Affiliated Hospital of Fujian Medical University. It was conducted following the Code of Declaration of Helsinki and was approved by the Ethics Committee. In this study, patients meeting the following inclusion criteria were included.

1. A pathology-confirmed diagnosis of PitNETs.
2. CECS or EES for primary or recurrent tumors.
3. Maximum diameter of ≥ 4 cm.
4. Significant intracranial tumor extension has at least one of the following features: (a) anterior progression toward the anterior skull base, (b) Posterior progression into the interpeduncular cistern, (c) Lateral progression into the middle temporal lobe or cranial fossa, (d)Superior extension reaching roof of the third ventricle and even to lateral ventricle, and/or the tumor breaches the sellar diaphragm or affects nearby neurovascular structures such as the circle of Willis, optic chiasm, hypothalamus, and the main cranial nerves (Fig. 1).

The excluded patients include those whose tumors respond to drugs, those with only expansive sphenoid/clivus tumors, those with severe dysfunction of vital organs, those with immune deficiency-related diseases, and those who are prone to infection due to long-term use of hormones, immunosuppressants, and other drugs, as well as those with other diseases such as malignant tumors and a short expected survival period.

Fifty patients met these criteria and were enrolled. Among these, 27 patients were treated by CECS, and 23 patients were treated by purely EES. The CECS procedures were performed by the same team of neurosurgeons (JCZ and SJP). The purely EES were performed by JCZ.

Data for tumor imaging features, maximum tumor diameter, and tumor infiltrate regions was collected by preoperative magnetic resonance imaging (MRI). Tumors were staged according to their parasellar and suprasellar extension by Knosp Steiner (KS) grading [13] and Hardy classification [14]. The extent of resection (EOR) was evaluated by volumetric analysis on MRI before and after surgery. The EOR was classified into 4°: gross total resection (GTR), no tumor residue was observed; Near total Resection (NTR), > 95% tumor tissue was removed or functional adenomas that achieved GTR but still had endocrine dysfunction; Subtotal Resection (STR), 75% to 95% tumor tissue was removed; Partial Resection (PR), less than 75% tumor tissue was removed.

All patients received endocrinological examination pre- and post-surgery, including growth hormone (GH), Cortisol, adrenocorticotrophic hormone (ACTH), prolactin (PRL), thyroid-stimulating hormone (TSH), luteinizing hormone (LH), follicle-stimulating hormone (FSH), testosterone (T), and estradiol (ES). The diagnosis of functioning adenoma was confirmed by clinical symptoms, imaging studies including MRI and computed tomography (CT), and endocrinological examination. Vision acuity (VA) and vision field (VF) tests were performed on all patients by ophthalmologists.

Perioperative complications such as intracranial infection, cerebrospinal fluid (CSF) leakage, new pituitary dysfunction, postoperative diabetes insipidus, oculomotor nerve paralysis, postoperative bleeding, vascular infarction, and mortality were retrieved from the database of the hospital or follow-up. The follow-up of the patients with an MRI and evaluation in the outpatient clinic at 3 months postoperatively, 6 months postoperatively, and once annually. Recurrence was defined as the regrowth of a residual tumor or the emergence of tumors detected on neuroimaging in patients who underwent GTR.

### CECS technique

**Fig. 2:**
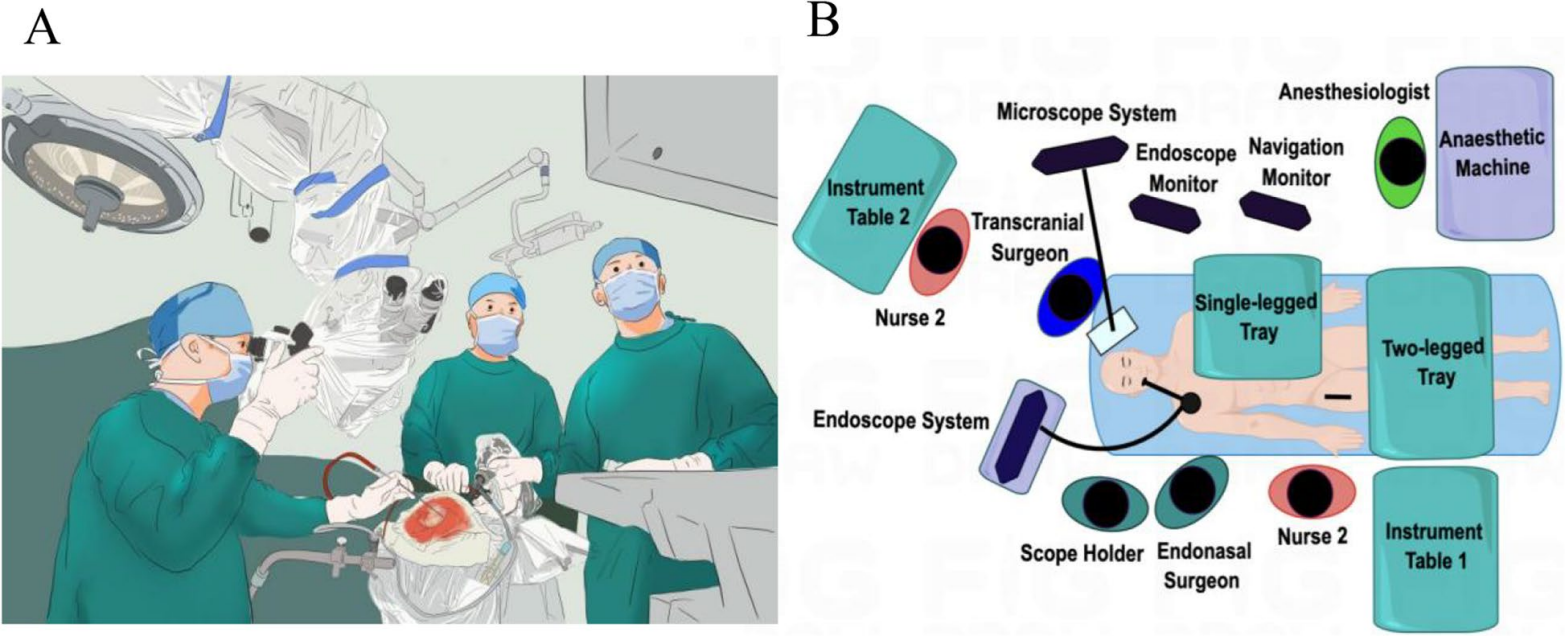
Schematic diagrams **A** and **B** of the setup for the two surgical teams for the CECS. Reprinted from 'How we do it: the double keyhole micro-endoscopic combined complex pituitary adenoma surgery', by Jianping Song, in *Acta neurochirurgica* vol, 2023, with permission

The CECS procedure involves two surgical teams: one performing EES and the other performing transcranial surgery. Additionally, transcranial surgery involves making clean incisions under a microscope, while endonasal surgery involves handling potentially contaminated incisions under an endoscope. Each team is led by an independent primary surgeon, assisted by support staff and nurses, and utilizes a separate set of surgical instruments. These two approaches are kept separate to prevent contamination. Usually, the EES surgeon stands on the right side of the patient and the transcranial surgeon stands on the head side of the patient (Fig. 2) [15]. A pterional approach, subfrontal approach, or supraorbital subfrontal eyebrow keyhole approach was used for performing transcranial surgery according to the suprasellar location of the tumor on MRI.

After anesthesia induction and endotracheal intubation, the patient is positioned supine with the head tilted 10° to the left, rotated 15° to the right, and extended 10° posteriorly. The head is secured with a head frame, and a neuronavigation system is used to assist in locating the tumor. The main components of the combined surgery process include the following three parts.

EES parts: the nasal cavity is disinfected with medical iodophor cotton patties, and adrenaline cotton wipes are used to constrict the nasal mucosa. A neuroendoscope is then inserted through the middle nasal meatus to locate and confirm the sphenoid sinus opening. A vascularized nasoseptal flap is prepared for skull base reconstruction. The sphenoid sinus is opened to fully expose the sella turcica. The opening of the sella turcica bone is expanded sideways toward the cavernous sinus, downward to the connection with the clivus, and upward to the tuberculum sellae. The dura mater is incised to expose the tumor, and a portion of the tumor is gently removed from the sella to reduce its size.

Transcranial surgery parts: according to the preoperative plan for the transcranial approach, a standard procedure is performed. Using the pterional approach as an example: The scalp and galea aponeurotica are incised, carefully stripping the temporalis while avoiding damage to the facial nerve. This reveals the Keyhole and pterional. Two or sometimes three burr holes in the skull are drilled, and a bone flap (approximately 5 × 6 cm) is created. The dura mater is suspended, and the incision is safeguarded with moist gauze and a film cover to prevent contamination. The dura is opened in a curvilinear fashion around the superior orbital fissure. Separating the Sylvian fissure, elevating the frontal lobe, and pulling apart the temporal lobe to expose the tumor extending upwards and establish the surgical corridor. The tumor’s capsule is meticulously separated from the nearby brain tissue and neurovascular structures, with a focus on preserving the capsule’s integrity when possible.

**Fig. 3:**
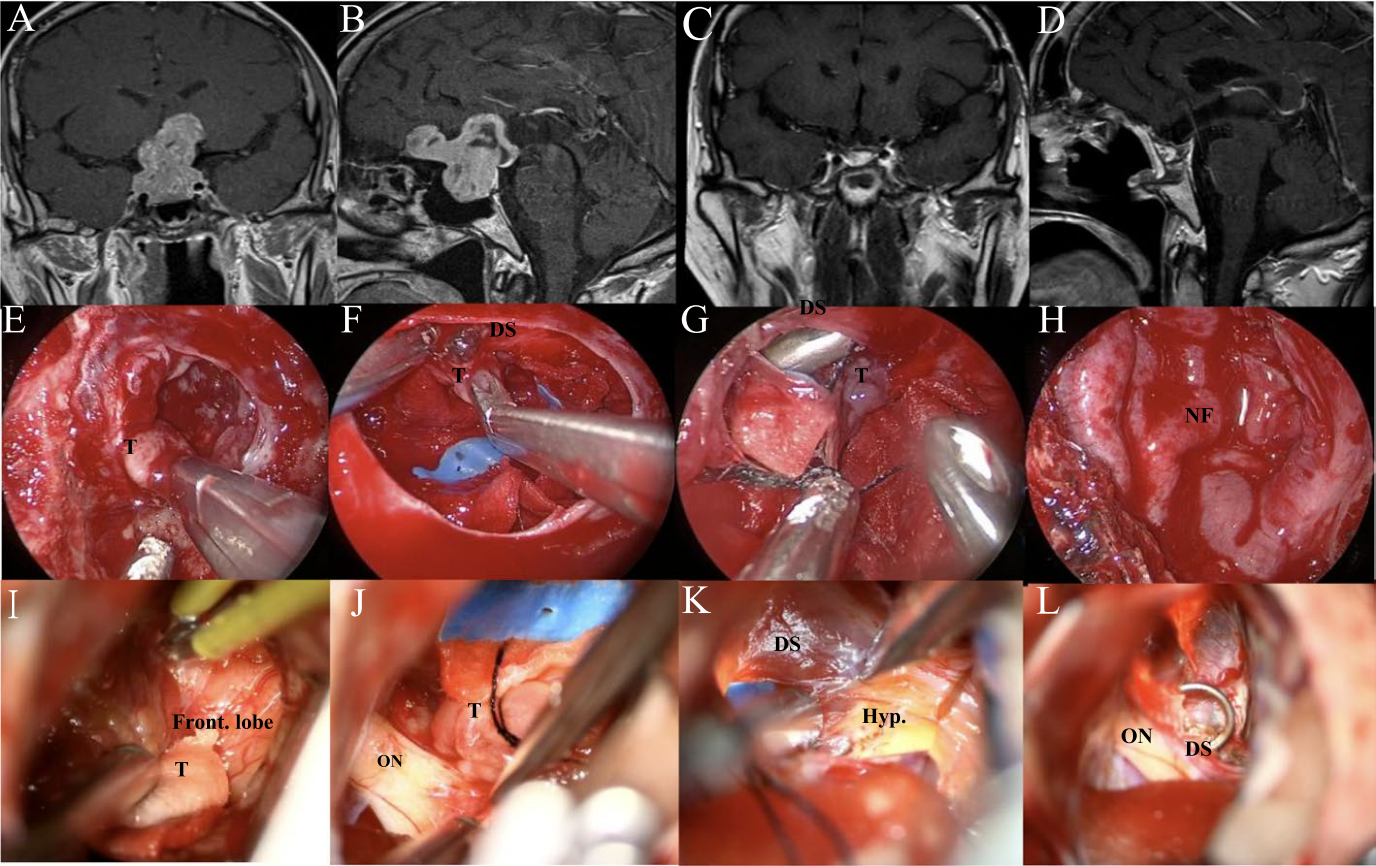
A 48-year-old male patient presented with visual loss and subsequently underwent CECS. **A**, **B** Preoperative MR image revealing GIPitNETs with significant anterior skull base and suprasellar extension. **C**, **D** Postoperative MR image showing GTR of the tumor. **E**–**H** Intraoperative images of patients who underwent surgery via the endoscopic endonasal approach. **E** Endscopic endonasal excision of the sellar portion of the tumor. **F** Excision of the tumor with diaphragma sellae adhesions. **G** Excision of the suprasaddle portion of the tumor pushed into the saddle by the transcranial surgeon. H Repair of the saddle base with a nasoseptal flap. **I**–**L** A simultaneous subfrontal approach was used. The tumor was dissected from adjacent lobe (**I**, **K**) and neurovascular (**J**) structures and was completely removed under direct intracranial observation or mechanically delivered into the sella to be removed during endonasal surgery (**L**). T, tumor; ON, optic nerve; DS, diaphragma sellae; Nf, nasoseptal flap; Front. lobes, frontal lobes; Hyp, hypothalamus

Combined surgery parts: following the anatomical dissection of the tumor and suprasellar neurovascular structures by the transcranial surgeon, cotton wipes were gently placed to push down the tumor tissue. Simultaneously, the endonasal surgeon continued to excise the displaced tumor tissue within the sella. This process was repeated iteratively. Finally, the endonasal surgeon, with the assistance of an endoscope, closely inspected for any remaining tumor within the sella, assessed the condition of the suprasellar vasculature and examined the ventricular system structure to confirm the absence of apparent tumor remnants and active bleeding. Meanwhile, the transcranial surgeon explored the area anterior to the suprasellar region anterior to the anterior skull base, posterior to the interpeduncular cistern, superiorly to the laminae terminalis, and laterally to the medial aspect of the temporal lobe, ensuring the absence of tumor remnants and active bleeding. Both surgical teams collaborated to reconstruct the sellar floor. Under transcranial direct vision, this approach allowed for preventing the overfilling of materials in the sella, which could potentially compress structures such as the optic nerve and hypothalamus. After the sellar floor repair was completed, each surgeon performed meticulous closure of the skull and nasal cavity, respectively (Fig. 3).

### Purely EES technique

**Fig. 4:**
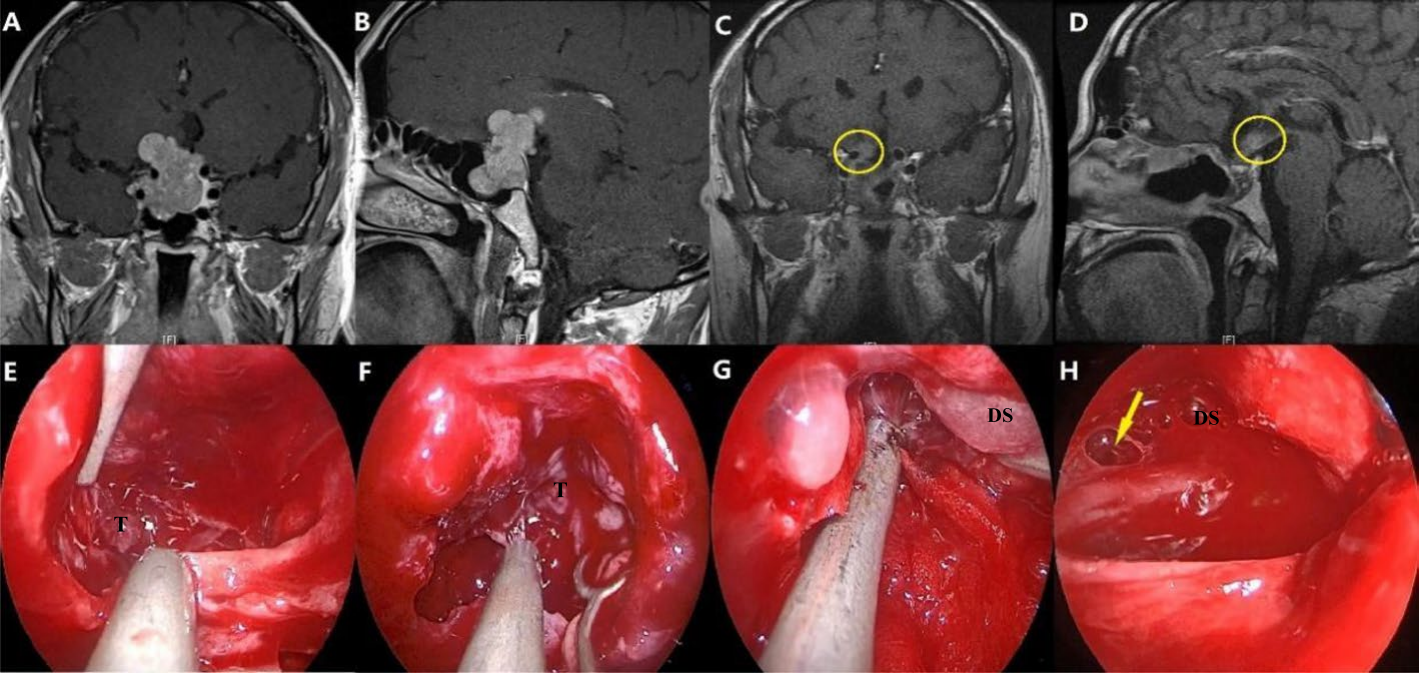
Example of a 43-year-old male patient with GIPitNETs who underwent EES. **A**, **B** Preoperative MR coronal and sagittal MR images showing GIPitNETs extending toward the suprasaddle, with the suprasaddle portion of the tumor extending upward to the right. **C**, **D** Three-month postoperative MR coronal and sagittal enhancements showing a residual tumor in the suprasaddle (yellow circle). **E** Removal of the intrasaddle portion of the tumor. **F** The part of the tumor adhering to the diaphragma sellae was removed. **G** Further upward removal of the suprasaddle portion of the tumor. **H** A breach in the right diaphragma sellae (yellow arrow) can be seen after resection of the suprasaddle portion of the tumor. In combination with postoperative MR images, the residual tumor should be a right-sided extension of the tumor through the diaphragma sellae. T, tumor; DS, diaphragma sellae

The initial surgical approach establishment and the reconstruction of the skull base are consistent with the endonasal surgical procedure in CECS. However, the difference lies in the fact that, after the removal of the intrasellar tumor, the removal of the suprasellar portion begins, Sometimes, it is necessary to enlarge the defect of the sella diaphragm to access and remove the tumor extending into the suprasellar region. If necessary, it may be required to expand the grinding of the sellar base bone in order to achieve the removal of the tumor adjacent to the sellar. In cases where there is a blind spot in the field of vision that cannot be reached with a 0° endoscope, a 30° or 70° endoscope is used for observation and surgical procedures (Fig. 4).

### Statistical analyses

Continuous variables are reported as mean ± SD or median with interquartile range (IQR), determined by the Shapiro–Wilk test. Categorical data are reported as counts and proportions in each group. The data between the groups were compared using the chi-square test (Fisher’s exact test where appropriate) for categorical variables or 2-tailed Student *t*-test (Mann–Whitney *U* test where appropriate) for continuous variables. IBM SPSS Statistics version 26.0 (IBM Corp.) were used to perform statistical analyses. *P* < 0.05 was considered statistically significant.

## Results

### Demographics

A total of 50 patients with GIPitNETs underwent surgery at The First Affiliated Hospital of Fujian Medical University. Among these, 27 patients were treated by CECS from September 2021 to May 2023 and 25 patients were treated by purely EES from March 2018 to May 2023. The patient demographics and clinical data are summarized in Table 1. The mean age in the CECS group was 50.5 ± 13.7 years, and in the EES group, it was 51.7 ± 16.8 years. There were 11 males (40.7%) and 16 females (59.3%) in the CECS group, while in the EES group, there were 15 males (65.2%) and 8 females (34.8%). The mean body mass index (BMI) was 25.7 ± 3.4 kg/m^2^ (CECS), and 24.4 ± 3.9 kg/m^2^ (EES). In the CECS group, there were 5 patients with hypertension (18.5%), 8 patients with diabetes (29.6%), and 8 patients (29.6%) were treated for recurrence. In the EES group, there were 5 patients with hypertension (21.7%), 3 patients with diabetes (13.0%), and 4 patients (17.4%) were treated for recurrence.

**Table 1:** Demographic and clinical data of the patients in the two groups

Parameters	Overall	CECS	EES	*P*
No. of patients	50	27	23	
Mean age, years	51.1 ± 15.01	50.5 ± 13.7	51.7 ± 16.8	0.772
Sex	–			0.084
Male (%)	26 (52.0%)	11 (40.7%)	15 (65.2%)	
Female (%)	24 (48.0%)	16 (59.3%)	8 (34.8%)	
Mean BMI, kg/m^2^	25.1 ± 3.6	25.7 ± 3.4	24.4 ± 3.9	0.218
Hypertension (%)	10 (20.0%)	5 (18.5%)	5 (21.7%)	0.777
Diabetes (%)	11 (22.0%)	8 (29.6)	3 (13.0%)	0.158
Recurrent (%)	12 (24.0%)	8 (29.6%)	4 (17.4%)	0.313
Course, months (IQR)	12 (3–24)	12 (3–36)	10 (3–12)	0.144
Clinical presentation				
Visual impairment (%)	46 (92.0%)	25(92.6%)	21(91.3%)	0.867
Bitemporal hemianopia (%)	44 (88.0%)	25 (92.6%)	19 (82.6%)	0.279
Headache (%)	20 (40.0%)	10 (37.0%)	10 (43.5%)	0.643
Preoperative hypopituitarism (%)	24 (48.0%)	17 (63.0%)	16 (69.6%)	0.623
Diabetes insipidus (%)	3 (6.0%)	1 (3.7%)	2 (8.7%)	0.459
Hydrocephalus (%)	11 (22.0%)	7 (25.9%)	4 (17.4%)	0.468
Pituitary apoplexy (%)	7 (14.0%)	4 (14.8%)	3 (13.0%)	0.857
Functioning PitNET (%)	5 (10.0%)	3 (11.1%)	2 (8.7%)	0.777

For the CECS group, symptoms were present from 7 days to 10 years before diagnosis. The most common symptoms were visual impairment (92.6%) and bitemporal hemianopia (92.6%), including two cases of blindness. A total of 10 patients (37.0%) had headaches, 17 patients (63.0%) had preoperative hypopituitarism, 1 patient (3.7%) experienced diabetes insipidus, 7 patients (25.9%) had hydrocephalus, and 4 patients (14.8%) suffered from tumor apoplexy. For the EES group, symptoms were present from 3 days to 9 years before diagnosis. Among them, 21 patients (91.3%) had visual impairment, and 19 patients (82.6%) had bitemporal hemianopia, including one case of blindness. Additionally, 10 patients (43.5%) experienced headaches, 16 patients (69.6%) had preoperative hypopituitarism, 2 patients (8.7%) had diabetes insipidus, 4 patients (17.4%) had hydrocephalus, and 3 patients (13.0%) suffered from tumor apoplexy. This study did not intervene in patients with preoperative hydrocephalus as their symptoms were mild and did not affect surgical tolerance. No recurrent hydrocephalus cases occurred during the follow-up.

Regarding tumor type, only 3 patients (11.1%) in the CECS group had a functioning PitNET, while 2 patients (8.7%) in the EES group had the same condition. Among them, one case in the CECS group was a PRL-secreting adenoma, while the remaining cases were GH-secreting adenomas. There were no statistically significant differences in demographics and basic clinical data between the two groups.

### Tumor imaging data

The overall tumor imaging data are presented in Table 2. The mean greatest tumor dimension was 4.82 ± 0.98 cm (CECS) and 4.53 ± 0.64 cm (EES). Tumors were staged according to their parasellar extension using KS grading, and in the CECS group, this included 2 (7.4%) grade I, 9 (33.3%) grade II, 8 (29.6%) grade III, and 8 (29.6%) grade IV tumors; in the EES group, there were 7 (30.4%) grade I, 5 (21.7%) grade II, 6 (26.1%) grade III, and 5 (21.7%) grade IV tumors. The suprasellar extension of the tumors was defined according to the Hardy classification, resulting in 8 (29.6%) grade III, 11 (40.7%) grade IV, and 8 (29.6%) grade V tumors in the CECS group, and 11 (47.8%) grade III, 6 (26.1%) grade IV, and 6 (26.1%) grade V tumors in the EES group. The details of tumor-infiltrating neighboring regions can be found in Table 2.

**Table 2:** Tumor imaging data of the patients in the two groups

Parameters	Overall	CECS	EES	*P*
Mean greatest tumor dimension (cm)	4.7 ± 0.8	4.8 ± 0.9	4.5 ± 0.6	0.207
Infiltrate regions (%)				
Sphenoid sinus	29 (58.0%)	19 (70.4%)	10 (43.5%)	0.055
Clivus	12 (24.0%)	8 (29.6%)	4 (17.4%)	0.313
Sella diaphragm	33 (66.0%)	19 (70.4%)	14 (60.9%)	0.480
Third ventricle	23 (46.0%)	12 (44.4%)	11 (47.8%)	0.811
Lateral ventricle	6 (12.0%)	3 (11.1%)	3 (13.0%)	0.834
Interpeduncular cistern	21 (42.0%)	9 (33.3%)	12 (52.2%)	0.179
Anterior skull base	30 (60.0%)	18 (66.7%)	12 (52.2%)	0.297
Parasella	18 (36.0%)	12 (44.4%)	6 (26.1%)	0.178
Circle of Willis	19 (38.0%)	12 (44.4%)	7 (30.4%)	0.309
KS grade (%)				0.177
Grade I	9 (18.0%)	2 (7.4%)	7 (30.4%)	
Grade II	14 (28.0%)	9 (33.3%)	5 (21.7%)	
Grade III	14 (28.0%)	8 (29.6%)	6 (26.1%)	
Grade IV	13 (26.0%)	8 (29.6%)	5 (21.7%)	
Hardy grade (%)				0.325
Grade III	19 (38.0%)	8 (29.6%)	11 (47.8%)	
Grade IV	17 (34.0%)	11 (40.7%)	6 (26.1%)	
Grade V	14 (28.0%)	8 (29.6%)	6 (26.1%)	

### Surgical results and perioperative complications

By analyzing the surgical records, in the CECS group, 17 patients (63.0%) were treated via a pterional approach, followed by 5 patients (18.5%) via subfrontal approaches, and 5 patients (18.5%) via supraorbital subfrontal eyebrow keyhole approaches in transcranial steps.

The difference in EOR between the groups reached statistical significance, with a higher proportion of GTR observed in the CECS group (66.7% vs. 13.0%, *p* < 0.0001, Table 3). Intraoperative blood loss (450 (300–1000) ml vs. 300 (150–500) ml, *p* = 0.002) and average operating time (7.3 ± 1.8 h vs. 4.3 ± 1.2 h, *p* < 0.0001) in the CECS group were significantly higher than in the EES group, showing a statistically significant difference. Hospital days (15 (14–21) days vs. 11 (7–20) days, *p* = 0.092) and postoperative hospital days (26 (22–32) days vs. 20 (14–29) days, *p* = 0.063) in the CECS group were longer, although no significant difference was found.

**Table 3:** Surgical results and perioperative complications of the patients in the two groups

Parameters	Overall	CECS	EES	*P*
EOR (%)				0.0001
GTR	21 (42.0%)	18 (66.7%)	3 (13.0%)	
NTR	19 (38.0%)	9 (33.3%)	10 (43.5%)	
STR	6 (12.0%)	0 (0%)	6 (26.1%)	
PR	4 (8.0%)	0 (0%)	4 (17.4%)	
Intraope blood loss, ml (IQR)	400 (200–650)	450 (300–1000)	300(150–500)	0.002
Operation time, h	5.93 ± 2.13	7.3 ± 1.8	4.3 ± 1.2	0.0001
Postop Hospital days, d (IQR)	15 (9–20)	15 (14–21)	11(7–20)	0.092
Hospital days, d (IQR)	24 (17.7–30)	26 (22–32)	20(14–29)	0.063
Periop complications (%)				
Intracranial infection	18 (36.0%)	9 (33.3%)	9 (39.1%)	0.670
CSF leakage	3 (6.0%)	1 (3.7%)	2 (8.7%)	0.459
New pituitary dysfunction	24 (48.0%)	14 (51.9%)	10 (43.5%)	0.555
Postoperative diabetes insipidus	15 (30.0%)	6 (22.2%)	9 (39.1%)	0.193
Oculomotor nerve paralysis	6 (12.0%)	2 (7.4%)	4 (17.4%)	0.279
Postoperative bleeding	21 (42.0%)	2 (7.4%)	15 (65.2%)	0.0001
Vascular infarction	3 (6.0%)	1 (3.7%)	2 (8.7%)	0.459
Periop mortality	1 (2.0%)	0 (0%)	1 (4.3%)	0.274

By contrast, EES significantly increased the risk of postoperative bleeding (7.4% vs. 65.2%,* p* < 0.0001). All other perioperative complications stratified by treatment modality are presented in Table 3, and no significant differences were observed in intracranial infection, CSF leakage, new pituitary dysfunction, postoperative diabetes insipidus, oculomotor nerve paralysis, and vascular infarction between the two groups. One patient in the CECS group died due to brain herniation caused by an acute cerebral infarction.

### Last follow-up outcomes

The median follow-up period was 14.8 (4.9–20.0) months in the CECS group and 34.0 (28.4–47.4) months in the EES group. In this study, Improved visual outcomes were more common in the CECS group, although no significant difference was found (77.8% vs. 47.8%, *p* = 0.108). In the CECS group, among the 3 cases of functioning PitNETs, 2 cases achieved biochemical remission. In the EES group, there were 2 cases of functioning PitNETs, with 1 case achieving biochemical remission. Statistical significance was not established due to the small sample size.

All 4 recurrent cases were from the EES group. Among them, all underwent reoperations, and three were treated with radiotherapy after the reoperations. In the CECS group, one acromegaly patient with Knosp Grade 4, who had a residual tumor within the cavernous sinus that could not be completely resected, underwent radiotherapy to control tumor progression. The patients in the CECS group had no tumor recurrence or reoperations, demonstrating a statistically significant difference compared to the EES group (Table 4).

**Table 4:** Last follow-up outcomes of the patients in the two groups

Parameters	Overall	CECS	EES	*P*
Follow-up, mos. (IQR)	21.9 (11.2–34.6)	14.8 (4.9–20.0)	34.0 (28.4–47.4)	0.0001
Biochemical remission	3 (3/5)	2 (2/3)	1 (1/2)	
Visual outcome (%)				0.108
Improved	32 (64.0%)	21 (77.8%)	11 (47.8%)	
Unchanged	17 (34.0%)	6 (22.2%)	11 (47.8%)	
Worsened	1 (2.0%)	0 (0%)	1 (4.3%)	
Recurrence (%)	4 (8.0%)	0 (0%)	4 (17.4)	0.024
Postop radiotherapy (%)	4 (8.0%)	1 (3.7%)	3 (13.0%)	0.225
Reoperations (%)	4 (8.0%)	0 (0%)	4 (17.4%)	0.024

## Discussion

Giant PitNETs are defined as a tumor with a maximum diameter greater than 4 cm [16, 17], but this alone does not fully indicate the surgical complexity. It's important to note that GIPitNETs extend widely in the intracranial compartment, representing a true surgical challenge because of their size, local invasiveness, irregular margins, and the involvement of the critical neurovascular structures. In view of this, radical removal rates are as low as 50% in numerous published surgical studies, with a greater risk of complications [3, 9, 18]. As of now, there is no consensus on the most effective surgical strategy for GIPitNETs.

The transcranial approach was considered the primary treatment choice for giant PitNETs by some neurosurgeons, considering that it is effective in removing tumors that extend beyond the sella [19, 20]. However, it has limited visualization of the intrasellar region [21, 22]. With the development of endoscopic endonasal surgery (EES), especially extended endonasal approaches, which provide clearer visibility of the sellar regions and have expanded the boundaries of endonasal approaches to include these tumors and the entire skull base [23, 24], this approach has been widely used in surgery for giant PitNETs [25–27]. Nevertheless, when the tumor exhibits excessive intracranial extension that is outside the visibility and maneuverability of the endoscopic endonasal route, the maneuvers for tumor removal become precarious, and the extent of resection is significantly reduced by purely EES [9, 27, 28].

Given this, some neurosurgeons recommend staged endonasal and craniotomy procedures [26, 29, 30]. On the contrary, some studies posit that partial debulking may elevate the risk of postoperative bleeding, leading to mass effect, and secondary surgery. Hence, it is recommended to aim for maximal tumor resection in the initial operation whenever possible [12, 31–34]. Recently, simultaneous combined endonasal and transcranial surgery, providing excellent exposure of the tumor and surrounding vital structures, has been proposed for the complete resection of GIPitNETs [8, 12, 25, 34–36]. However, there is a lack of studies comparing its effectiveness and risks with the current mainstream treatment of GIPitNETs using purely EES. Further investigation is needed to evaluate the differences between these two approaches.

### The extent of resection comparison and CECS advantage

In this study, the similar baseline characteristics of patients and radiological features of the tumor in both groups make the comparison of surgical outcomes and perioperative complications feasible. The study demonstrated that using the CECS, a 66% vs. 13% gross tumor removal was achieved compared with the EES group (*p* < 0.0001), and the superior results of CECS over the reported rates (46% and 50%) in two other studies [12, 33]. Based on our experience, CECS offers several advantages for achieving maximum resection of GIPitNETs. These advantages include, first, endoscopic endonasal surgery can remove most midline tumors, and parts of the extended tumor located outside the endoscopic view can be removed or pushed into the endoscopic operative view from the transcranial approach. Second, parts of the tumor extend into the frontal lobe or into the third ventricle, outside the transcranial view, and the range of endonasal surgical procedures can be observed under the endoscope, guiding transcranial surgeons during the tumor removal process. Third, when neurovascular structures are encased in the tumor above the sellar, purely endoscopic endonasal surgery may have difficulty dissecting the tumor from neurovascular structures, such a dissection procedure can be performed safely with transcranial surgery. All of the aforementioned advantages of CECS can significantly increase the total resection rate of GIPitNETs.

### Visual and biochemical remission camparison

Although a higher proportion of improved visual outcomes was observed in the CECS group, the difference did not reach statistical significance (77.8% vs. 47.8%, *p* = 0.108). One possible explanation is that the surgical approach does not affect visual symptom improvement in treating giant PitNETs, This is because even partial tumor removal can alleviate pressure on the optic nerve, leading to symptom relief [37, 38]. So, despite a higher tumor removal rate in the CECS, there's no major difference in visual symptom relief.

Functional giant PitNETs are less common, constituting approximately 30% of all giant PitNETs [18]. In our study, which included 5 cases (10%), we did not observe a significant difference in post-surgery outcomes between the two approaches, possibly due to the limited number of cases. Furthermore, it is important to note that different types of functional PitNETs exhibit varying behaviors, which can have an impact on patient outcomes. Additional data on functional giant PitNETs are required for a more comprehensive analysis of the two surgical methods. In simple terms, achieving biochemical remission can be challenging as long as hormonally active tumor tissue remains. The primary goal is to safely remove as much tumor tissue as possible, even if complete removal is difficult. This is where the CECS approach excels.

### Perioperative complications comparison and impact on surgery

Several studies have reported that incomplete resection following endonasal surgery for giant PitNETs puts patients at risk of residual suprasellar tumor tissue hemorrhage, postoperative bleeding, and edema, resulting in increased mass effect, compression of the optic pathway, apoplexy, and acute hydrocephalus, with documented cases of fatalities as a result [21, 28, 31, 32, 39]. With the simultaneous transcranial approach, the tumor can be dissected from surrounding neurovascular structures, observed for safer resection, significantly reduced the tumor volume, increased the rate of total resection, and decreased the occurrence of postoperative bleeding of the residual tumor. This is consistent with our research findings, as the postoperative bleeding occurrence rate in the EES group is significantly higher than in the CECS group (7.4% vs. 65.2%, *p* < 0.0001). For postoperative bleeding patients, we do immediate monitoring and assessment to initially determine the bleeding impact. Head CT scans are closely rechecked to clarify the bleeding range changes and surrounding tissue compression. For those with little bleeding and no neurological deterioration, conservative treatments like hemostatic drugs and strict blood pressure control are given. Surgical intervention is taken when the bleeding is severe (with worsened neurofunctional disorders or ineffective conservative treatment). Generally, the standard pterional or subfrontal approach is used to clear the hematoma. Under our management, the majority of patients with postoperative bleeding had relatively favorable results in both the short-term postoperative recovery and the subsequent follow-up. In the EES group, one of the cases experienced a rapid decline in vision due to residual suprasellar tumor tissue hemorrhage and had to undergo a second surgery to remove the hematoma. In another case, vascular infarction following postoperative suprasellar subarachnoid hemorrhage leads to a stroke, followed by cerebral edema and brain herniation, ultimately resulting in death. Such events were not observed in the CECS group. Additionally, in this study, the rates of other complications such as CSF leakage, new pituitary dysfunction, postoperative diabetes insipidus, oculomotor nerve paralysis, vascular infarction, and perioperative mortality were similar between the two groups. This suggests that CECS is a safe and viable surgical approach.

Some studies suggest that a combined approach also has disadvantages of greater invasiveness, including a longer operation time and a higher risk of postoperative intracranial infection, along with complications related to both endonasal and transcranial surgery [8, 12, 33]. Indeed, in our research report, the CECS group had longer operation time (7.3 ± 1.8 h vs. 4.3 ± 1.2 h, *p* < 0.0001), and greater surgical trauma, necessitating a longer hospital stay (26 (22–32) days vs. 20 (14–29) days,* p* = 0.063) for recovery compared to the EES group. However, we reported a similar rate of postoperative intracranial infection between the groups (33.3% vs. 39.1%, *p* = 0.670), and no cases of infection were observed after the use of this combined approach in the fewer case reports by Kuga (4 cases) [8] and Inoue (6 cases) [12]. There are currently no large-sample reports on postoperative intracranial infection rates in GIPitNETs, and we are the first to report this. Postoperative intracranial infection is influenced by various factors, including the defect of the sella diaphragm and the degree of cerebrospinal fluid leakage during surgery [40–42]. Further data collection and analysis are needed to study the impact of the surgical approach on postoperative intracranial infection.

Certainly, surgical experience is indispensable for favorable surgical outcomes. Surgeons with extensive experience possess superior coordination, a strong ability to identify anatomical landmarks and avoid risks, and have fewer postoperative complications. The learning curve of CECS has distinct phases, evolving from basic endoscopic skills to complex cranial cavity operations. It is necessary to master endoscopic techniques and the principles of cranial base neurosurgery to make accurate judgments during surgical procedures. The complexity of CECS poses challenges. Continual education and the like are indispensable for enhancing its efficacy and safety.

### Follow-up outcomes

Recurrence caused by the residual tumor was observed in 4 cases (17.4%) in the EES group. Upon reviewing the imaging data, all 4 recurrent patients had residual tumors in the suprasellar area and underwent second surgeries through transcranial procedures. One patient achieved complete tumor removal, while three patients with Knosp Grade 4 still had remnants in the cavernous sinus, leading to additional radiotherapy. During the follow-up period (ranging from 4.9 to 20 months), no tumor recurrences were observed in the CECS group. These results are consistent with Kuga1's follow-up of 5–59 months and Inoue's follow-up of 6–41 months [8, 12]. This result supports the idea that CECS is more effective for achieving GTR for tumors extending to suprasellar space, while EES may leave residual suprasellar tumors, especially when residual tumors are close to the optic apparatus that is a relative contraindication to radiotherapy [43], thereby increasing the need for additional surgical treatments. However, due to the relatively late introduction of the CECS in our center, the significantly different follow-up durations (14.8 (4.9–20.0) months vs. 34.0 (28.4–47.4) months, *p* < 0.0001) make it challenging to compare long-term outcomes. Further follow-up data are needed to support our study.

## Limitations

This study was retrospective. Despite rigorous screening of patients in accordance with the inclusion and exclusion criteria, it lacked randomization and double-blinding, giving rise to a certain selection bias. Additionally, the selection of surgical methods was influenced by multiple factors such as patients’ desires, the surgeon’s experience, and the degree of development of surgical techniques. In this study, cases in the EES group commenced in January 2018, while the CECS technique was only implemented in our center as of January 2021. Changes in the surgeon's notions and proficiency in surgical operations during this period potentially affected surgical efficacy, complications, and prognosis. Furthermore, due to the novelty of CECS surgery and the limitation of the follow-up time, it is challenging to visualize the long-term follow-up data of this group (especially concerning tumor recurrence). We will formulate a plan for further in-depth investigation as the follow-up time extends in the future, thereby facilitating readers to understand the current research status and its subsequent development direction comprehensively and thoroughly. Secondly, the number of cases of complex GIPitNETs in our center was relatively insufficient, and the data were limited. This might also be one of the reasons why some study indicators failed to reach statistical significance. The differences in efficacy and risks between EES and CECS in treating complex GIPitNETs are of great significance for guiding the treatment options of GIPitNETs and the development of combined approach techniques. In the future, more case data and large-scale multicenter controlled studies are requisite to provide stronger evidence for the benefits and risks of both.

## Conclusions

For GIPitNETs, CECS can achieve a significantly higher rate of GTR compared to EES. While CECS led to longer operation times and hospital stays, it demonstrated similar rates of the risks of postoperative complications such as intracranial infection, CSF leakage, new pituitary dysfunction, postoperative diabetes insipidus, and vascular infarction were comparable to those in the EES group. Additionally, CECS can reduce the risk of postoperative bleeding, thereby decreasing the occurrence of some severe consequences such as compression of the optic pathway and apoplexy. Furthermore, tumor recurrence and reoperation at the last follow-up were significantly more often encountered in patients treated with EES. Our research results confirm that CECS is a safe and effective surgical treatment approach for GIPitNETs.

## Supplementary Information

Supplementary Material 1.

## Data Availability

Not applicable.
