# Supplementary figures and images for: Giant and irregular pituitary neuroendocrine tumors surgery: comparison of simultaneous combined endoscopic endonasal and transcranial and purely endoscopic endonasal surgery at a single center
Source: Chin Neurosurg J. 2025 Feb 3;11:3. doi: 10.1186/s41016-025-00389-4 (PMC11789305; doi:10.1186/s41016-025-00389-4)

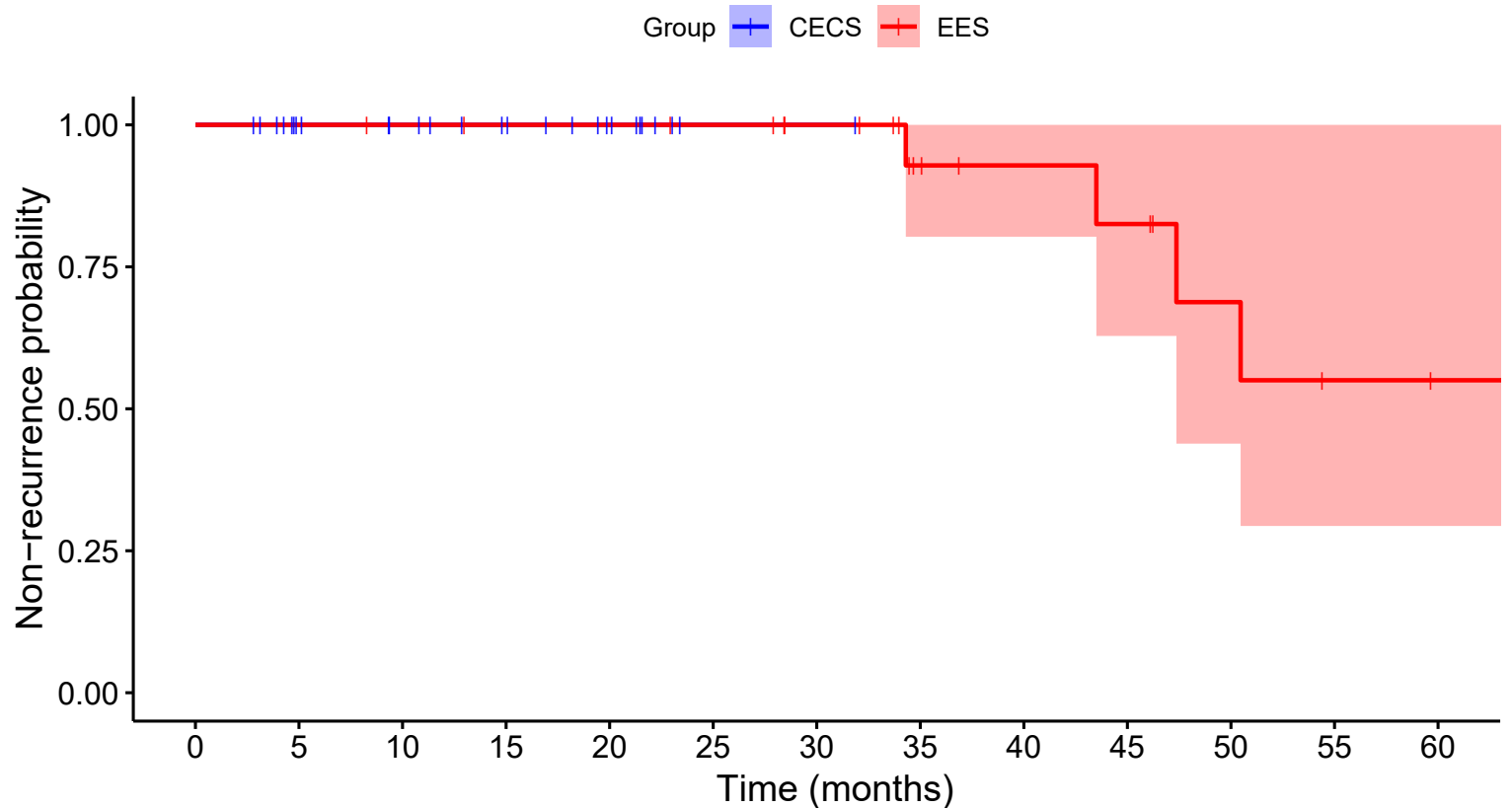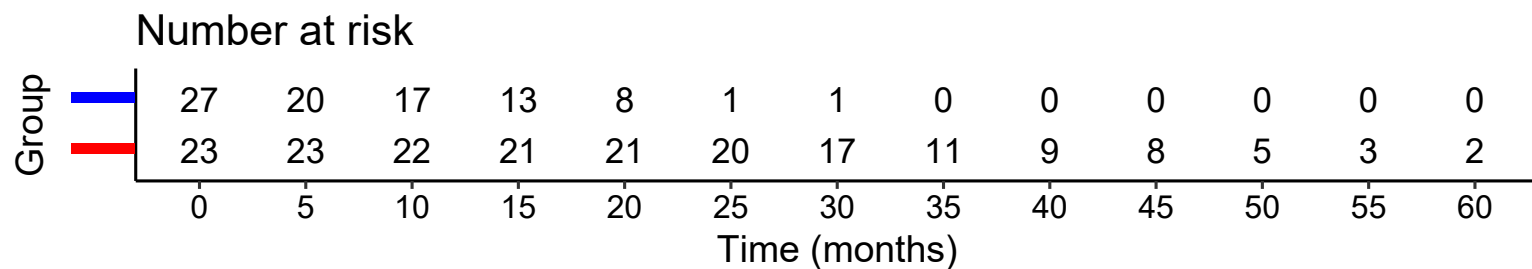

Supplement: Supplementary file 1 — Supplementary Material 1. [file 41016_2025_389_MOESM1_ESM.pdf]
